# Chemical Investigation and Screening of Anti-Proliferative Activity on Human Cell Lines of Pure and Nano-Formulated Lavandin Essential Oil

**DOI:** 10.3390/ph13110352

**Published:** 2020-10-29

**Authors:** Elisa Ovidi, Valentina Laghezza Masci, Anna Rita Taddei, Patrizia Paolicelli, Stefania Petralito, Jordan Trilli, Fabio Mastrogiovanni, Antonio Tiezzi, Maria Antonietta Casadei, Pierluigi Giacomello, Stefania Garzoli

**Affiliations:** 1 Department for the Innovation in Biological, Agrofood and Forestal Systems, Tuscia University, 01100 Viterbo, Italy; eovidi@unitus.it (E.O.); laghezzamasci@unitus.it (V.L.M.); fabiomastrogiovanni85@gmail.com (F.M.); antoniot@unitus.it (A.T.); 2 High Equipment Centre, Tuscia University, 01100 Viterbo, Italy; artaddei@unitus.it; 3 Department of Drug Chemistry and Technology, Sapienza University, 00185 Roma RM, Italy; patrizia.paolicelli@uniroma1.it (P.P.); stefania.petralito@uniroma1.it (S.P.); jordan.trilli@uniroma1.it (J.T.); mariaantonietta.casadei@uniroma1.it (M.A.C.); pierluigi.giacomello@uniroma1.it (P.G.)

**Keywords:** lavandin essential oil, antiproliferative activity, HS-GC/MS analysis, nanoemulsion

## Abstract

Lavandin essential oil (LEO), a natural sterile hybrid obtained by crossbreeding *L. angustifolia × L. latifolia,* is mainly composed by active components belonging to the family of terpenes endowed with relevant anti-proliferative activity, which can be enhanced by proper application of nanotechnology. In particular, this study reports the chemical characterization and the screening of the anti-proliferative activity on different human cell lines of pure and nano-formulated lavandin essential oil (EO). LEO and its formulation (NanoLEO) were analyzed by HS/GC-MS (Headspace/Gas Chromatography-Mass Spectrometry) to describe and compare their chemical volatile composition. The most abundant compounds were linalool and 1,8-cineole (LEO: 28.6%; 27.4%) (NanoLEO: 60.4%; 12.6%) followed by α-pinene (LEO: 9.6%; NanoLEO: 4.5%), camphor (LEO: 6.5%; NanoLEO: 7.0%) and linalyl acetate (LEO: 6.5%; NanoLEO: 3.6%). The cytotoxic effects of LEO and NanoLEO were investigated on human neuroblastoma cells (SHSY5Y), human breast adenocarcinoma cells (MCF-7), human lymphoblastic leukemia cells (CCRF CEM), human colorectal adenocarcinoma cells (Caco-2) and one normal breast epithelial cell (MCF10A) by the MTT (3-(4,5-Dimethylthiazol-2-yl)-2,5-Diphenyltetrazolium Bromide)-assay. Caco-2, MCF7 and MCF10A normal cells resulted more resistant to the treatment with LEO, while CCRF-CEM and SHSY5Y cells were more sensitive. The antiproliferative effect of LEO resulted amplified when the essential oil was supplied as nanoformulation, mainly in Caco-2 cells. Scanning and transmission electron microscopy investigations were carried out on Caco-2 cells to outline at ultrastructural level possible affections induced by LEO and NanoLEO treatments.

## 1. Introduction

Plants provide a large amount of secondary metabolites, some of which are used in their original or semisynthetic form, to treat different human pathologies [1]. Among the secondary metabolites, essential oils, obtained by classical or by more modern methods of extraction from different parts of aromatic plants such as flowers, leaves, rhizomes, seeds, wood, fruits and bark [2,3], are a complex mixture rich in volatile molecules which varies in quantity and quality according to exogenous and endogenous factors [4].

Essential oils (EOs) are mainly composed of monoterpene, sesquiterpene hydrocarbons and their oxygenated derivatives and possess numerous biological activities [3,5,6,7,8]. Lavender essential oils are characterized by a wide variety of volatile constituents [9]. In Lavandula species, such as *L. angustifolia*, *L. intermedia*, *L. stoechas* and *L. latifolia,* the main class of components are the oxygenated monoterpenes whose biological activities were also studied [10]. Lavender oils are commonly used in aromatherapy and in numerous applications for therapeutic and cosmetic purposes [11]. Their biological activities were investigated and antioxidant [12,13,14,15], anxiolytic and antidepressive [16,17,18], antifungal and bactericidal [6,19], cytotoxic [20,21], anti-inflammatory [22] and analgesic [23], autophagy and apoptosis induction properties [8,24] have been reported. The chemical composition of the essential oils is conserved in Lavandula species and differs only for the proportion of the components. Good but different antioxidant activities were also revealed for different cultivars of lavandin and lavender EOs by more assays such as β-carotene/linoleic acid bleaching (lipid peroxidation inhibition), DPPH (2,2-diphenyl-1-picrylhydrazyl) % and ABTS (2,2′-azino-bis(3-ethylbenzothiazoline-6-sulfonic acid) % + (radical scavenging activity) [12].

Further investigations on lavender essential oils showed antitumor properties on different cell lines and apoptotic and necrosis induction mechanisms were observed [25,26,27,28]. Monoterpenes were demonstrated to possess an antitumor activity [29] as it has been reported for linalool [30,31,32], 1,8 cineole [33] and linalyl acetate [34].

Unfortunately, pharmaceutical application of lavender essential oils is greatly limited by their poor physico-chemical properties, including their limited water solubility, which requires the production of an adequate formulation in order to overcome poor biopharmaceutical properties an fully exploit their therapeutic potential [35]. Among all the suitable formulation strategies, aqueous nanoemulsions are gaining increasing interest for the effective delivery of essential oils thanks to the easy fabrication and handling as well as the limited manufacturing costs. They are ultrafine isotropic dispersed systems of two non-miscible liquids, generally consisting of an oily phase dispersed in an aqueous one, in the form of nanometer-sized droplets stabilized by an interfacial film of surfactant. Most of the physical and pharmaceutical properties of nanoemulsions are a consequence of the small size of the dispersed globules. In particular, the large specific surface area of the colloidal dispersion promotes solubility and permeation of delivered active compounds across biological membranes, thus resulting in improved bioavailability and pharmacological efficacy.

The nanometer-size of the dispersed globules is also responsible for the kinetic stability of this colloidal dispersion respect to conventional emulsion systems. In fact, nanoemulsions avoid issues such as coalescence during long-term storage as Brownian motions of the nanometric droplets are able to overcome gravitational separation forces [36]. While nanoemulsions are kinetically stable systems, they are thermodynamically unstable and their formation can be achieved with the application of an external energy input that allows exceeding the energy gap between the separated phases and the colloidal system. Depending on how the external energy input is provided, the methods for the fabrication of nanoemulsion can be divided in high and low-energy ones. Most of the low-energy techniques used for the preparation of nanoemulsions involve heating or evaporation phases of the solvent, which can lead to the volatilization and/or degradation of the components of an essential oil altering its chemical composition. Therefore, it will be crucial to characterize the essential oil chemical profile before and after formulation to verify eventual qualitative and quantitative differences.

In this paper the solvent displacement method was used for the fabrication of an o/w nanoemulsion as delivery system for lavandin essential oil (LEO). It involves a solvent evaporation step, which may lead to volatilization and/or degradation of EO, thus influencing its final composition and functional properties. For this purpose, headspace (HS) extraction system coupled with a GC/MS was performed [37]. This technique results the most suitable because it excludes the use of solvents for sample preparation and avoids any pretreatment.

The antiproliferative activity of LEO on several human tumor cell lines including human neuroblastoma cells (SHSY5Y), human breast adenocarcinoma cells (MCF-7), human lymphoblastic leukemia cells (CCRF CEM), human colorectal adenocarcinoma cells (Caco-2) and one normal breast epithelial cell (MCF10A), was investigated. The chemical composition and the activity exerted by the oil formulated in nanoemulsion (NanoLEO) on the same cell lines were also investigated to compare the biological effects with pure LEO. For this purpose, scanning and transmission electron microscopy investigations (SEM and TEM) were also carried out to outline at ultrastructural level possible affections induced by LEO and NanoLEO treatments.

## 2. Results

### 2.1. LEO and NanoLEO Chemical Analysis

LEO and NanoLEO samples were investigated by headspace-gas chromatography/mass spectrometry (HS-GC/MS) analysis. Twenty-three components were identified and listed in Table 1. In particular, in LEO, acetic acid hexyl ester and terpinolene were missing while in NanoLEO camphene, β-pinene and trans-β-ocimene have not been found. Linalool and 1,8-cineole were most abundant components of both LEO (28.6 ± 0.04%; 27.4 ± 0.01%) and NanoLEO (60.4 ± 0.03%; 12.6 ± 0.01%) followed by α-pinene (LEO: 9.6 ± 0.01%; NanoLEO: 4.5 ± 0.02%), camphor (6.5 ± 0.01%; 7.0 ± 0.01%) and linalyl acetate (LEO: 6.5 ± 0.01%; NanoLEO: 3.6 ± 0.01%). These four analytes belong to the terpene family.

**Figure 1:**
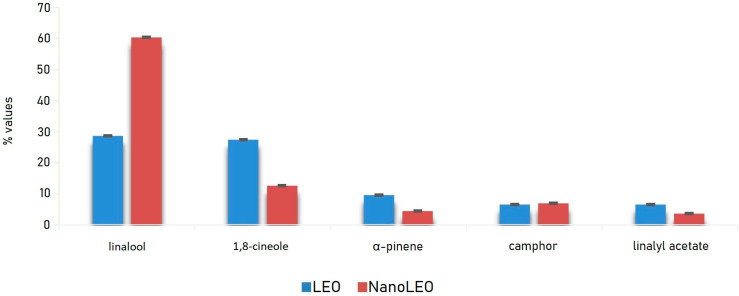
Bar graph of the values (%) of major compounds detected in lavandin essential oil (LEO) and nanoemulsion of lavandin essential oil (NanoLEO). *p* < 0.05 according to ANOVA test.

The graph below (Figure 1) relating to the trend of the main components, identified by HS/GC-MS (Headspace/Gas Chromatography-Mass Spectrometry) analysis, shows clearly that they were detected in higher percentages in the vapor phase of LEO than in that of NanoLEO with the exception of linalool which, on the contrary, represented the component with the highest relative percentage in NanoLEO.

### 2.2. Nanoemulsion Characterization

LEO was formulated in nanoemulsion using a solvent displacement–evaporation technique, which did not require a high-energy input, as emulsification occurred spontaneously when the organic phase containing LEO was poured at room temperature into the aqueous phase. After evaporation of the organic solvents used, a nanometer-sized o/w emulsion with a narrow size distribution was formed. The formulation displayed a mean hydrodynamic diameter of 302.0 ± 2.4 nm, a PdI of 0.128 ± 0.001 and a ζ potential of +47.2 ± 1.6 mV, as determined by DLS and ELS (Dynamic Light Scattering and Electrophoretic Light Scatterin) respectively. The morphology, dimensions and homogeneity of NanoLEO were also evaluated by negative stain TEM, which evidenced the formation of spherical globules and confirmed the results from the DLS analysis.

### 2.3. Cytotoxicity Test (MTT)

To evaluate the cytotoxic effects of pure LEO and NanoLEO, a cell viability assay (MTT) was carried out on five different cell lines. The assay was performed both in a dose dependent and time dependent manner. 

As reported in Table 2, the EC_50_ values obtained after LEO treatments showed, for CCRF-CEM, SHSY5Y and Caco-2 cells, a dose dependent and not time dependent effects ranging from 1.30 × 10^−2^ ± 0.09 × 10^−2^% for 24 h to 1.25 × 10^−2^ ± 0.08 × 10^−2^% for 72 h in CCRF-CEM, from 1.42 × 10^−2^ ± 0.08 × 10^−2^% for 24 h to 1.97 × 10^−2^ ± 0.10 × 10^−2^% for 72 h in SHSY5Y cells and from 13.52 × 10^−2^ ± 1.97 × 10^−2^% for 24 h to 12.45 × 10^−2^ ± 0.99 × 10^−2^% for 72 h for Caco-2 cells. The two breast cells lines have shown a slight time dependent effect ranging from 3.62 × 10^−2^ ± 0.76 × 10^−2^% after 24 h to 4.53 × 10^−2^ ± 0.68 × 10^−2^% after 72 h of treatment for the breast cancer cell line (MCF7) and from 6.73 × 10^−2^ ± 1.04 × 10^−2^% after 24 h to 3.47 × 10^−2^ ± 0.06 × 10^−2^% after 72 h of treatment for the normal breast epithelial cell (MCF10A).

The NanoLEO treatments demonstrated both a dose an time dependent effects: ranging from 0.57 × 10^−2^ ± 0.08 × 10^−2^% for 24 h to 0.87 × 10^−2^ ± 0.05 × 10^−2^% for 72 h for CCRF-CEM cells; from 0.64 × 10^−2^ ± 0.05 × 10^−2^% for 24 h to 0.44 × 10^−2^ ± 0.02 × 10^−2^% for 72 h for SHSY5Y; from 1.79 × 10^−2^ ± 0.53 × 10^−2^% (24 h) to 1.58 × 10^−2^ ± 0.09 × 10^−2^% for 72 h for Caco-2 cells; from 1.73 × 10^−2^ ± 0.10 × 10^−2^% for 24 h to 1.35 × 10^−2^ ± 0.21 × 10^−2^% for 72 h for the MCF7 cells; from 0.90 × 10^−2^ ± 0.14 × 10^−2^% for 24 h to 0.25 × 10^−2^ ± 0.14 × 10^−2^% for 72 h for the MCF10A cells (Table 2).

**Figure 2:**
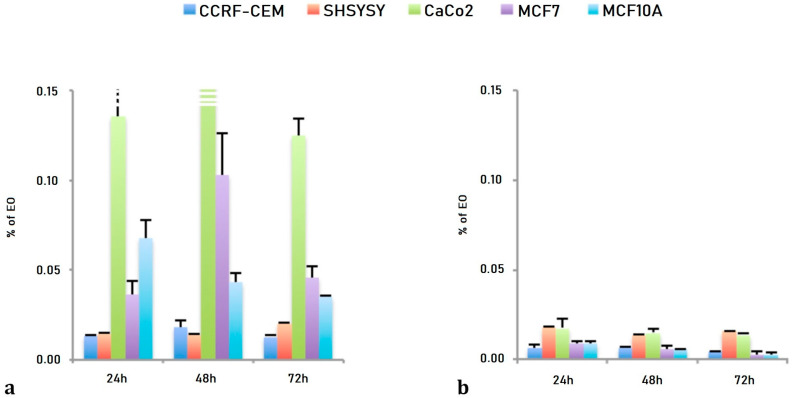
Bar graph of the EC_50_ values, expressed as percentage of essential oil (EO) obtained after 24 h, 48 h and 72 h of treatment. (**a**) lavandin essential oil (LEO) and (**b**) nanoemulsion of lavandin essential oil (NanoLEO). *p* < 0.05 according to ANOVA test.

The effects of both LEO and NanoLEO were different between the cell lines considered (Figure 2). In Figure 2a the EC_50_ values obtained after three different incubation times with LEO were represented and the Caco-2, the MCF7 and the MCF10A cells were more resistant to the treatment while the CCRF-CEM and the SHSY5Y cells resulted more sensitive. The EC_50_ values obtained after the treatment with NanoLEO were reported in Figure 2b. The effects of LEO were slightly amplified in nanoformulation for CCRF-CEM and SHSY5Y; otherwise the MCF7, MCF10A and mostly CaCo-2 cells were even more affected by NanoLEO respect to LEO treatments. The DMSO control and the nanoemulsion control did not inhibit the proliferation of all the cell lines investigated.

### 2.4. Statistical Analysis

The data from all independent experiments were expressed as means ± standard deviation (SD). Statistical analysis was performed using a one-way ANOVA test with a Stat-Plus software (AnalystSoft ©2009) with the threshold of significance set at *p* < 0.05.

### 2.5. Ultrastructure Investigation by Electron Microscopy

**Figure 3:**
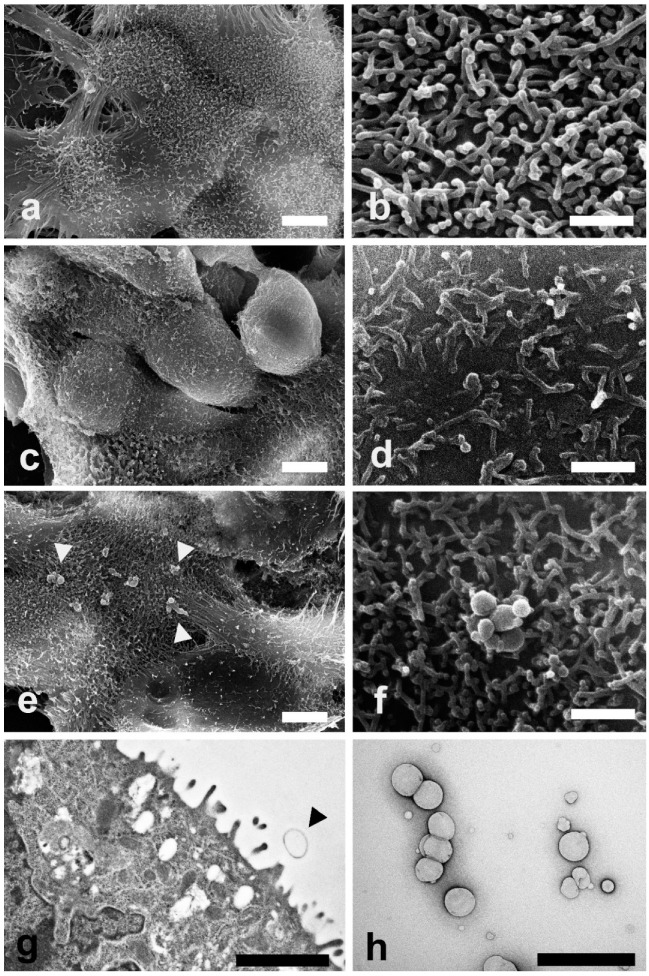
Electron microscopy investigations were carried out to evaluate morphological changes induced by LEO and NanoLEO treatments (**a**) Scanning electron microscopy (SEM) micrograph of untreated Caco-2 cells at low magnification; (**b**) SEM micrograph of cellular protrusions of untreated Caco-2 cells membranes; (**c**) SEM micrograph of Caco-2 cells treated for 4 h with LEO; (**d**) A particular of the LEO treated Caco-2 cells surface by SEM; (**e**) SEM micrograph of NanoLEO treated Caco-2 cells where nanoemulsions particles were observed (arrow heads); (**f**) Nanoemulsion globules with a round shape; (**g**) transmission electron microscopy (TEM) micrograph of nanoemulsion treated cells. Arrow heads evidence a particle; (**h**) Negative staining technique shows NanoLEO globules with a different diameter. Bars = (**a**,**c**,**e**) 5 µm; (**b**,**d**,**f**,**h**) 1 µm; (**g**) 2µm.

SEM and TEM investigations were carried out on Caco-2 cells exposed to LEO and NanoLEO treatments for 4 h. In Figure 3a control cells (untreated cells) show their typical shape with the surface characterized by the presence of numerous and regularly distributed protrusions (Figure 3b). In LEO treated cells, a smoother cell surface was observed (Figure 3c) and morphological changes in cell protrusions occurred appearing shorter and not uniformly distributed (Figure 3d). No differences were evidenced on the surface in NanoLEO treated cells comparing to the control (Figure 3e) and cell surface showed protrusions with a normal length and morphology. Numerous roundish particles were observed (arrow heads) on the cell surface of NanoLEO treated cells (Figure 3f). TEM on ultrathin sections confirmed the presence of roundish shaped particles on the cell surfaces which appeared defined by a single layer membrane (Figure 3g, arrow heads). Figure 3h showed negatively stained nanoemulsion with different droplets size observed by TEM.

**Figure 4:**
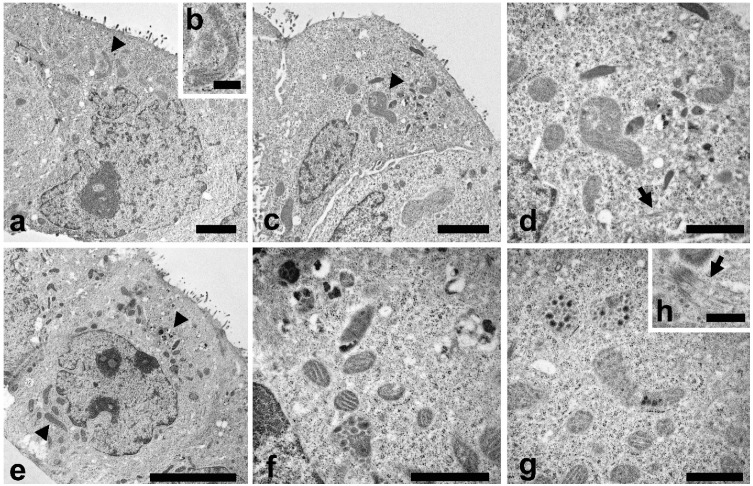
TEM investigations were carried out to evaluate morphological changes induced by LEO and NanoLEO treatments. (**a**) TEM micrograph of untreated Caco-2 cells at low magnification with mitochondria well visible (arrowhead); (**b**) Normal mitochondrion; (**c**) TEM micrograph of Caco-2 cells treated for 4 h with LEO; altered mitochondria (arrowheads); (**d**) A particular of the LEO cell where altered mitochondria were visible; apoptotic microtubule network were observed (arrow); (**e**) TEM micrograph of NanoLEO treated Caco-2 cells (arrowheads: mitochondria); (**f**,**g**): Two particulars of the NanoLEO treated Caco-2 cells; with altered mitochondria; (**h**) apoptotic microtubule network were observed (arrow); ). Bars = (**a**) 2 µm; (**b**,**h**) 500 nm; (**c**) 2 µm; (**d**,**f**,**g**) 1 µm; (**e**) 5 µm.

TEM analysis were also carried out to study the cell ultrastructure (Figure 4). In control cells a typical cytoplasmic organization can be observed (Figure 4a), with a regular nucleus, endoplasmic reticulum, Golgi apparatus and numerous normal shaped mitochondria (Figure 4a, arrowhead). A higher magnification of mitochondrion is reported in Figure 4b.

In LEO treated cells (Figure 4c) some mitochondria showed modifications in their features. Mitochondria were swollen and with altered cristae structure (arrowhead) and a more electron dense matrix was observed. A higher magnification (Figure 4d) put in evidence mitochondria morphological changes and the presence of bundles of microtubules inside the cytoplasm (arrow). NanoLEO treatments determined deep alterations in the mitochondria (Figure 4e), as reported in numerous cells where these organelles resulted enlarged and with various degree of modifications as the presence of electrodense granule, translucent space and longitudinally oriented or disorganized cristae (Figure 4f,g). In the cytoplasm, bundles of microtubules were observed (Figure 4h, arrow). Moreover, some cells characterized by shrinkage, retraction of pseudopods and plasma membrane blebbing were observed (Supplementary Figure S1, SEM micrograph of apoptotic Caco-2 cells).

## 3. Discussion

Nowadays lavender essential oil is still investigated to better clarify its biological properties. The anticancer activities of essential oils from numerous plant species and their constituents have been studied in different human cell lines in vivo and in vitro as reviewed by Gautam et al. [38]. Once essential oils have penetrated into the cell membrane, they act through different mechanisms by which essential oils carry out their anticancer action including apoptosis, cell cycle arrest, antimetastatic and antiangiogenic, increased levels of reactive oxygen and nitrogen species, DNA repair modulation and others. Russo et al. [39], reported that different essential oils showed anticancer activities in vitro and in vivo models underlining among them those whose activities are due to the phytocomplex. Clear evidences were reported on apoptosis induction mechanism exerted by essential oils: *Melissa officinalis* essential oil induced apoptosis of glioblastoma multiforme cells [40], *Salvia milthiorriza* treatment reduced the proliferation of HepG2 hepatoma cells, changing their morphology and inducing cell death by apoptosis [41], *Artemisia annua* essential oil induced apoptosis in SMMC-7721 hepatocarcinoma cells [42] and essential oil of the conifer tree *Tetraclinis articulata* showed pro apoptotic activity in human melanoma and ovarian cancer cell lines [43]. In our previous paper, LEO treatment has been reported to induce apoptosis on HL60 human leukemia cells and, among the main LEO compounds, both terpinen-4-ol and linalyl acetate possess antiproliferative activity [28].

In the present paper, LEO and its nanoformulation NanoLEO have been analyzed by HS/GC-MS technique to describe the volatile chemical profile of their vapor phase. The results reported in Table 1 highlighted that linalool (LEO: 28.6% ± 0.04; NanoLEO: 60.4% ± 0.03) and 1,8-cineole (LEO: 27.4% ± 0.01; NanoLEO: 12.6% ± 0.01) were the most abundant components. Linalool showed cytotoxic activity on C32 cells line [44] and human leukemia and lymphoma cell lines [45]. In particular, linalool induced apoptosis of numerous leukemia cells by upregulation of p53 and cyclin-dependent kinase inhibitors [30]. Furthermore, other minor components in association, such as linalyl acetate, alpha-terpineol and camphor also found in LEO and NanoLEO, have been reported to be able to induce inhibition of the growth of the human colon cancer cell lines (HCT-116 p53+/+ and p53−/−) [46]. The cytotoxicity of 1,8-cineole was well described in more papers. For example, Hayes et al. [47] reported a moderate cytotoxic action on Hep G2, HeLa, MOLT-4, K-562 and CTVR-1 cell lines while Cha and colleagues [48] found that this compound, induced apoptosis of KB cells through caspase activation.

LEO and NanoLEO have been then tested on human neuroblastoma cells (SHSY5Y), human breast adenocarcinoma cells (MCF7), human lymphoblastic leukemia cells (CCRF CEM), human colorectal adenocarcinoma cells (Caco-2) and on normal breast epithelial cells (MCF10A) in order to compare their biological activities. The screening of the antiproliferative activities for LEO and NanoLEO was carried out by MTT assay and the results revealed that the effects of LEO and NanoLEO were in a dose- and not time-dependent manner and different among the investigated cell lines (Figure 2). Concerning LEO, the EC_50_ values showed that Caco-2, MCF7and MCF10A cells were more resistant to the treatment while the CCRF-CEM and the SHSY5Y cells were more sensitive. It was interesting to observe how the antiproliferative effect of LEO was amplified when the essential oil was supplied in nanoformulation (NanoLEO). Such differences were highly evident in the more resistant cell lines where the affection level was consistently higher after treatment with nanoformulated samples. Also, in the CCRF-CEM and SHSY5Y, the effects of LEO were amplified in nanoformulation although not so strongly.

Several studies on the effects of *L. angustifolia* extracts and the essential oil carried out on different cancer cell lines confirmed the observed cytotoxic properties of Lamiaceae EOs and their potential role as anticancer drugs. Tayarani-Najaran et al. [25] reported high antiproliferative activity of both *L. angustifolia* extract and essential oil and apoptotic mechanism induction in Hela and MCF-7 cells by EtOH and n-hexane extract. Furthermore, Nikoli’c et al. [21] found antiproliferative activity on Hela and A549 cells, with EC_50_ values of 80.62 ± 1.04 µg/mL and 88.90 ± 1.71 µg/mL respectively. Gezici and collaborators [27] have also demonstrated the effects of lavender EOs at low concentrations and at a minimum exposure time. Moreover, studies on prostate cancer cells exhibited EC_50_ values of 0.199% ± 0.026% for DU145 cells and 0.037% ± 0.011% (*v*/*v*) for PC-3 cells, confirming the potent cytotoxic activity of lavender EOs [34]. It has been reported that *L.* × *intermedia* showed cytotoxic effects on human breast cancer cells MCF-7 [49] and the cytomorphological modification could represent the activation of a programmed cell death. Studies on the effects of *L. angustifolia* extracts and the essential oil on Hela and MCF-7 reported of an antiproliferative action although an apoptotic mechanism induction was not demonstrated [25].

Nanotechnology has been shown to be a suitable strategy for cancer treatment and nanoemulsions might be the solution for different types of cancer (colon, ovarian, prostate, leukemia, breast, lung and melanoma). These formulations solve water-solubility problems, provide specific targeting to cancer cells and reduce tumor growth [50]. As reported in the studies on MCF 7 cells treated with *Nigella stativa* essential oil [51], nanoemulsions were tested to improve the effects of essential oils. In this case, a reduced cell viability and an alteration of nuclear morphology in a dose- and time-dependent manner was relieved. *Zataria multiflora* incapsulated essential oil was investigated in breast cancer cells and an enhanced in vitro anticancer activity with respect to the corresponding essential oil was observed [52]. Nanoemulsions based ginger and frankincense essential oils can ameliorate the apoptotic effects of mitomycin C on HeLa cervical cancer cells and MCF-7 breast cancer cells [53].

The employment of a hydrophobic core also allows the encapsulation of drugs. Liu et al. [54] reported that the quinolone alkaloid evodiamine formulated in nanoemulsion was capable of making sensitive cancerous A549 cells thanks to improved cellular uptake.

In our investigation, Caco-2 cell line cytotoxicity test showed a relevant difference in EC_50_ values of LEO and NanoLEO treatments and the cancer colorectal cell line resulted much more sensitive to the nanoemulsion respects to not formulated LEO. In this view, Caco-2 cell line was further investigated by SEM and TEM to determine possible morphological alterations. 

It has been reported that treatments with essential oils from four lavender species on Caco-2 cells determine an alteration of the cellular surface which become smooth with tiny microvilli [55]. Our SEM observations (Figure 3), showed alterations of the plasma membrane protrusions in LEO treated cells; the protrusions appeared shorter and not uniformly distributed. Few nanoemulsions particles were observed on the cell surfaces because of the 60 nm sections. The particles size, observed by the negative staining procedure, reveal a correspondence with the previous defined range size.

Due to the nanometer size, generally in the range of 10–500 nm, nanoemulsions, are able to enhance the activity of EOs since they allow a better and deeper tissue penetration and an easier cellular uptake. Moreover, they allow control and modulation of the release of active ingredients on the target site [36].

The cellular uptake of nanoemulsions may occur through different pathways, such as macropinocytosis, clathrin-mediated endocytosis, caveolae-mediated endocytosis and clathrin- and caveolae-independent endocytosis [56]. In particular, the mechanism of uptake of nanoemulsions has been reported to vary appreciably due to differences in emulsifiers, surface properties, particle shape and size [57]. About this last point, more studies reported that increased cellular uptake is linked to decreased droplet size [58,59]. Endocytosis of emulsions is a complex mechanism and more detailed information about cellular uptake and transport mechanism of lipid-based nanoemulsions are currently unavailable and further studies are required.

In the present study some evidences (Figure 3) showed morphological alterations in the cells after LEO and NanoLEO treatments. Mitochondrial morphology resulted to be deeply altered; mitochondria appeared to be swollen, with longitudinally rearranged or disorganized cristae and with more electron dense matrix mainly in NanoLEO rather than in LEO treatment. The loss of mitochondrial membrane integrity with the release of cytochrome-c and other proapoptotic factors in the cytosol and the remodeling of the cristae membrane is the first morphological alteration of the apoptotic process [60,61].

In NanoLEO treated cells and in minor extension in LEO treated cells, microtubule bundles were observed. Microtubule bundles, known as apoptotic microtubule networks, play particularly important functions in maintaining the plasma membrane integrity [62] and the dispersion of cellular and nuclear fragments [63] during execution apoptosis.

Taken together the morphological alterations particularly evident in NanoLEO treated cells, such as mitochondria modifications and the presence of apoptotic microtubules networks and the previous results of LEO treatment on HL60 cells [28], we can hypothesize that LEO and more efficiently NanoLEO treatments induce apoptotic cell death.

In conclusion, by the HS-GC/MS chemical analyses, we relieved a loss of some components and consequently a slight change in the volatile profile of the essential oil following its formulation in nanoemulsion. This change in the chemical composition, should lead to a reduction on the antiproliferative activity. On the contrary, we observed an enhanced effect on all the cell lines tested and in particular on the Caco-2 cell line. Therefore, considering the above, we can assume that differences evidenced in the behavior and activity of LEO, with respect to NanoLEO, may be attributed to the formulation which may have favored cellular uptake of essential oil.

## 4. Materials and Methods

### 4.1. Materials

Pure commercial *Lavandula* × *intermedia* (lavandin) “Grosso” essential oil (LEO) (IT BIO 007 D86K, lot number MGL01/19) obtained from steam distillation, was directly provided by Azienda Podere dell’Arco (Viterbo, Lazio Region, Italy).

Soybean phosphatidylcholine (Phospholipon90; SPC) from Lipoid and LabrafacTM lipophile WL 1349 (medium-chain triglycerides of caprylic and capric acids) were kindly gifted by AVG and Gattefossé, respectively. Benzalkonium chloride, ethanol and acetone were obtained from Sigma-Aldrich (Darmstadt, Germany).

### 4.2. Gas Chromatography-Mass Spectrometry (GC-MS) Analysis

Essential oil, pure and formulated, was analyzed by a gas chromatograph directly coupled to a mass spectrometer (MS) Perkin Elmer Clarus 500 model (Waltham, MA, USA). The GC was equipped with a Restek Stabilwax (fused-silica) polar capillary column. Helium was used as carrier gas at a flow rate of 1 mL/min The injector was set to a 280 °C and the oven temperature program was as follows: isothermal at 60 °C for 5 min, then ramped to 220 °C at a rate of 5 °C min^−1^ and finally isothermal at 220 °C for 20 min. Front detector was an Electro Impact-Mass Spectrometer (EI-MS), mass spectra were recorded at 70 eV (EI) and were scanned in the range 40–400 m/z. Ion source and the connection parts temperature was 220 °C. The back detector was a FID (Flame Ionization Detector) maintained at 250 °C. The injector split ratio was 1:20. The GC-TIC (Total Ion Chromatogram) mass spectra were obtained by the TurboMass data analysis software (Perkin Elmer). The LEO constituents were identified by comparison of their linear retention indices (LRIs), (relative to C8–C30 aliphatic hydrocarbons, Ultrasci injected in the column at the same operating conditions described above) with the available retention data in the literature. Further identification of all components was made by matching their mass spectra with those stored in the Wiley and NIST 02 mass spectra libraries database. GC-FID analysis was performed under the same experimental conditions using the polar column as described for the GC-MS measurements. Relative percentages of all identified components, were obtained by peak area normalization from GC-FID chromatograms without the use of an internal standard or correction factors and expressed in percentages. All analyses were repeated twice.

### 4.3. Head Space GC-MS Analysis

The volatile composition of essential oil pure and formulated were performed by a Perkin Elmer Headspace Turbomatrix 40 (Waltham, MA, USA) autosampler connected to GC-MS. To optimize the headspace procedure for the determination of volatile organic compounds (VOCs), parameters such as equilibration time and temperature were optimized. The gas phase of the sealed vials was equilibrated for 20 min at 60 °C and was followed immediately by compound desorption into GC injector in splitless mode.

### 4.4. Nanoemulsion Preparation

The nanoemulsions were prepared following a procedure reported in Garzoli et al. [37]. The method followed for the nanoemulsion preparation was the solvent displacement technique, which involves the spontaneous emulsification of an organic phase added to an aqueous phase under magnetic stirring. More specifically, the organic phase consisted of 100 µL of LEO, 30 mg of SPC and 3 mg of benzalkonium chloride dissolved in a solvent mixture composed of 9.5 mL of acetone and 0.5 mL of ethanol. The addition of the organic phase to 10 mL of deionized water, maintained under magnetic stirring, led to the diffusion of the organic solvents and the immediate and spontaneous formation of the nanoemulsion, as evidenced by the milky appearance of the mixture. The organic solvents were removed by evaporation under reduced pressure until a final volume of 10 mL was achieved and the obtained formulation was labelled as Nano LEO. A control nanoemulsion was prepared with the same procedure but using Labrafac as the oily phase.

### 4.5. Physicochemical Characterization of Nanoemulsions

Hydrodynamic diameter (Z-average, nm), polydispersity index (PdI) and zeta potential (mV) were measured by Dynamic Light Scattering (DLS) and Electrophoretic Light Scattering (ELS) measurements with a Zetasizer Pro (Malvern Instruments Ltd., Malvern, Worcestershire, UK). The DLS and ELS techniques used a photon correlator spectrometer equipped with a 4 mW He/Ne laser source operating at 633 nm. All measurements were performed in non-invasive back scattering (scattering angle of 173°) and were thermostatically controlled at 25 °C. The samples were appropriately diluted with demineralized water before analysis.

### 4.6. MTT Test

The cytotoxic effects of LEO and its nano formulation, NanoLEO, were investigated by the MTT-assay. The assay was carried out on four different cancer cell lines: human neuroblastoma cells (SHSY5Y. American Type Culture Collection^®^ CRL-2266™), human breast adenocarcinoma cells (MCF7. ATCC^®^ HTB-22™), human lymphoblastic leukemia cells (CCRF CEM. ATCC^®^ CCL-119™), human colorectal adenocarcinoma cells (Caco-2. ATCC^®^ HTB-37™) and one normal breast epithelial cells (MCF10A. ATCC^®^ CRL-10317™).

2 × 10^4^ cells/well in complete culture medium were seeded in a 96-well micro plate 24 h before treatments. The SHSY5Y and MCF7 cells were cultured in DMEM F-12 (Dulbecco’s Modified Eagles Medium: nutrient mixture F-12) supplemented with 10% Fetal Bovine Serum (FBS), 1% glutamine and 1% penicillin-streptomycin. The CCRF CEM cells were cultured in RPMI-1640 (Roswell Park Memorial Institute medium) supplemented with 10% FBS, 1% glutamine and 1% penicillin-streptomycin. The Caco-2 cells were cultured in DMEM supplemented with 10% FBS, 1% glutamine, 1% sodium pyruvate, 1% non-essential amino acids and 1% penicillin-streptomycin. The MCF10A cells were cultured in DMEM F-12 supplemented with 100 ng/mL cholera toxin, 20 ng/mL epidermal growth factor (EGF), 0.01 mg/mL insulin, 500 ng/mL hydrocortisone and 5% Horse Serum (HS), 1% glutamine and 1% penicillin-streptomycin; before being seeded into the 96-well micro plate the culture medium has been deprived of EGF and the HS reduced to 2%. All the cell lines were maintained at 37 °C in a humidified 5% CO_2_ condition.

Ten 2-dilutions (0.1% to 1.95 × 10^−4^%) of essential oil were used and incubated for three different times. DMSO and a nanoemulsion prepared using Labrafac (a compound not having any cytotoxic effect as alone), as the oily phase and stabilized with benzalkonium chloride and SPC, at the 0.1% were used as control. Vinblastine was used as positive control (ten 2-dilutions from 0.25 µM to 0.48 × 10^−3^ µM).

After 24, 48 and 72 h, the medium containing treatments was removed and 100 µL of MTT solution (0.5 mg/mL) was added to each well and incubated in the dark at 37 °C for 3 h. The formazan crystals were dissolved in 100 µL of DMSO and the absorbance measured at 570 nm. The concentration at which a substance exerts half of its maximal response values (EC_50_) was calculated by using AAT Bioquest, Inc. (Sunnyvale, CA 94085, USA) (5 November 2019).

### 4.7. Electron Microscopy

For the electron microscopy analysis, 1 × 10^5^ Caco-2 cells were seeded onto a 24-well plate inserts and incubated for 24 h in appropriate culture conditions before treatments with Leo and NanoLEO at EC_50_ concentrations.

After 4 and 24 h, the cells were collected in tubes, washed with PBS and fixed with 4% paraformaldehyde and 5% glutaraldehyde, pH 7.2, in a 0.1 M cacodylate buffer for 1 h at 4 °C [64]. After rinsing overnight in the same buffer, samples were post-fixed in 1% osmium tetroxide in a cacodylate buffer for 1 h at 4 °C. After two washings in the same buffer, samples were dehydrated in a graded ethanol series.

For Scanning Electron Microscopy (SEM), cells were dried by the critical point method using CO_2_ in a Balzers Union CPD 020, sputter-coated with gold in a Balzers MED 010 unit and observed by a JEOL JSM 5200 electron microscope (Jeol Ltd., Tokyo, Japan).

For Transmission Electron Microscopy (TEM), samples were fixed and dehydrated as described above and embedded in an Epon mixture resin. Thin sections (50–70 nm) were cut with Reichert Ultracut (Leica Microsystems, Wetzlar, Germany) and LKB Nova ultramicrotomes (LKB Vertriebs GmbH, Vienna, Austria) using a diamond knife, collected on copper grids, stained with uranyl acetate and lead citrate and observed by a JEOL 1200EX II electron microscope.

For negative staining, the droplets of sample suspensions (10 µL) were placed on formvar-carbon coated grids and allowed to adsorb for 60 s. Excess liquid was removed gently touching the filter paper. The adsorbed specimen was then processed for negative-staining, by first washing the specimen grid on a drop of negative stain (2% uranyl acetate in distilled water), blotting and repeating this step once more, this time leaving the specimen grid for 60 seconds on a new drop of negative stain solution. Samples were observed at a JEOL 1200 EX II electron microscope. Micrographs were acquired by the Olympus SIS VELETA CCD camera equipped the iTEM software.

## 5. Conclusions

In this study, the chemical volatile composition of *Lavandula* × *intermedia* essential oil pure and encapsulated in nanoemulsion was characterized by HS-GC/MS technique and their antiproliferative activity investigated on different cell lines. The effects of LEO and NanoLEO occurred in a dose- and not time-dependent manner and in some of the tested cell lines NanoLEO formulation showed higher antiproliferative activity respect to LEO. Electron microscopy observations showed mitochondria modifications and apoptotic microtubules networks in LEO and more efficiently NanoLEO treatments, probably due to an apoptotic process. The mechanism of permeation of LEO and NanoLEO into the cells has not yet been established and it could represent an area of future investigation.

Since, to our knowledge, no study on comparative antiproliferative activity of LEO vs. NanoLEO has been conducted so far, this work is the first contribution for a better knowledge of the effectiveness of lavandin formulated oil compared to the pure essential oil.

## Figures and Tables

**Table 1:** Chemical composition (%) of lavandin essential oil (LEO) and NanoLEO.

N°	Component ^1^	LRI^lit 2^	LRI ^3^	LEO Area (%)	NanoLEO Area (%)
1	α-pinene	1021	1018	9.6 ± 0.01	4.5 ± 0.02
2	camphene	1065	1058	4.4 ± 0.01	-
3	β-pinene	1105	1101	3.3 ± 0.15	-
4	limonene	1198	1192	3.6 ± 0.02	0.6 ± 0.02
5	1,8-cineole	1209	1201	27.4 ± 0.01	12.6 ± 0.01
6	(Z)-β-ocimene	1237	1232	0.5 ± 0.04	1.4 ± 0.01
7	acetic acid hexyl ester	1275	1271	-	0.5 ± 0.03
8	trans-β-ocimene	1276	1274	0.4 ± 0.02	-
9	terpinolene	1282	1281	-	0.4 ± 0.01
10	o-cymene	1287	1291	1.7 ± 0.01	-
11	propanoic acid, 2-methyl-hexyl ester	1335	1330	0.3 ± 0.01	0.5 ± 0.02
12	1-octen-3-yl-acetate	1401.9	1401	0.6 ± 0.02	0.8 ± 0.01
13	hexyl butanoate	1410	1412	0.6 ± 0.04	-
14	linalool oxide	1423	1426	1.6 ± 0.02	1.4 ± 0.01
15	1-octen-3-ol	1458	1462	0.7 ± 0.01	0.3 ± 0.01
16	camphor	1507	1501	6.5 ± 0.02	7.0 ± 0.01
17	linalool	1537	1538	28.6 ± 0.04	60.4 ± 0.03
18	linalyl acetate	1553	1550	6.5 ± 0.01	3.6 ± 0.01
19	lavandulyl acetate	1584	1579	0.8 ± 0.01	-
20	terpinen-4-ol	1603	1601	1.5 ± 0.02	2.9 ± 0.02
21	lavandulol	1662	1665	0.3 ± 0.02	0.3 ± 0.01
22	α-terpineol	1675	1678	0.3 ± 0.02	1.3 ± 0.02
23	borneol	1717	1720	0.8 ± 0.02	1.5 ± 0.01
Total (%)				100.0	100.0

^1^ Elution order on polar column; ^2^ Linear Retention indices from literature; ^3^ Linear Retention indices measured on polar column.

**Table 2:** LEO and NanoLEO EC_50_ values obtained by dose-dependent MTT ((3-(4,5-Dimethylthiazol-2-yl)-2,5-Diphenyltetrazolium Bromide) assay after 24, 48 and 72 h of treatments. The values are expressed as mean ± SD. Vinblastine was used as positive control ^1^. *p* < 0.05 according to ANOVA test.

Cell Line	Time	EC_50_ % of EO (Mean ± SD)	
		LEO	NanoLEO
CCRF-CEM	24 h	1.30 × 10^−2^ ± 0.09 × 10^−2^	0.57 × 10^−2^ ± 0.08 × 10^−2^
	48 h	1.80 × 10^−2^ ± 0.37 × 10^−2^	0.47 × 10^−2^ ± 0.08 × 10^−2^
	72 h	1.25 × 10^−2^ ± 0.08 × 10^−2^	0.87 × 10^−2^ ± 0.05 × 10^−2^
SHSY5Y	24 h	1.42 × 10^−2^ ± 0.08 × 10^−2^	0.64 × 10^−2^ ± 0.05 × 10^−2^
	48 h	1.35 × 10^−2^ ± 0.08 × 10^−2^	0.62 × 10^−2^ ± 0.05 × 10^−2^
	72 h	1.97 × 10^−2^ ± 0.10 × 10^−2^	0.44 × 10^−2^ ± 0.02 × 10^−2^
Caco2	24 h	13.52 × 10^−2^ ± 1.97 × 10^−2^	1.79 × 10^−2^ ± 0.53 × 10^−2^
	48 h	24.12 × 10^−2^ ± 0.60 × 10^−2^	1.33 × 10^−2^ ± 0.28 × 10^−2^
	72 h	12.45 × 10^−2^ ± 0.99 × 10^−2^	1.58 × 10^−2^ ± 0.09 × 10^−2^
MCF-7	24 h	3.62 × 10^−2^ ± 0.76 × 10^−2^	1.73 × 10^−2^ ± 0.10 × 10^−2^
	48 h	10.25 × 10^−2^ ± 2.33 × 10^−2^	1.47 × 10^−2^ ± 0.20 × 10^−2^
	72 h	4.53 × 10^−2^ ± 0.68 × 10^−2^	1.35 × 10^−2^ ± 0.21 × 10^−2^
MCF-10A	24 h	6.73 × 10^−2^ ± 1.04 × 10^−2^	0.90 × 10^−2^ ± 0.14 × 10^−2^
	48 h	4.28 × 10^−2^ ± 0.51 × 10^−2^	0.60 × 10^−2^ ± 0.02 × 10^−2^
	72 h	3.47 × 10^−2^ ± 0.06 × 10^−2^	0.25 × 10^−2^ ± 0.14 × 10^−2^

^1^ EC_50_ values of Vinblastine: CCRF-CEM 24.11 ± 4.02 nM; SHSY5Y 1.92 to ± 0.14 nM; Caco2 3.74 × 10^3^ ± 0.13 × 10^3^; MCF-7 2.62 × 103 ± 0.38 × 10^3^ nM; MCF-10 ≥ 250 × 10^3^ nM.

## Supplementary Materials

The following are available online at https://www.mdpi.com/1424-8247/13/11/352/s1. Figure S1: SEM micrograph of apoptotic Caco-2 cells.
