# Supplementary material for: Chemical Investigation and Screening of Anti-Proliferative Activity on Human Cell Lines of Pure and Nano-Formulated Lavandin Essential Oil
Source: Pharmaceuticals (Basel). 2020 Oct 29;13(11):352. doi: 10.3390/ph13110352 (PMC7692866; doi:10.3390/ph13110352)
Supplement: Supplementary file 1 [file pharmaceuticals-13-00352-s001.pdf]

Supplementary Material

Chemical investigation and screening of anti-proliferative activity on human cell lines of pure and nano-formulated lavandin essential oil

Elisa Ovidi [1], Valentina Laghezza Masci [1], Anna Rita Taddei [2], Patrizia Paolicelli [3], Stefania Petralito [3], Jordan Trilli [3]; Fabio Mastrogiovanni [1], Antonio Tiezzi [1], Maria Antonietta Casadei[3], Pierluigi Giacomello [3], Stefania Garzoli [3]*

[1] Department for the Innovation in Biological, Agrofood and Forestal Systems, Tuscia University, Viterbo, Italy; laghezzamasci@unitus.it (V.L.M.); fabiomastro85@gmail.com (F.M.) antoniot@unitus.it (A.T.); eovidi@unitus.it (E.O.)

[2] High Equipment Centre, Tuscia University, 01100 Viterbo, Italy; artaddei@unitus.it (A.R.T.)

[3] Department of Drug Chemistry and Technology, Sapienza University, Rome, Italy; stefania.garzoli@uniroma1.it (S.G.); pierluigi.giacomello@uniroma1.it (P.G.); patrizia.paolicelli@uniroma1.it (P.P.); stefania.petralito@uniroma1.it (S.P.); mariaantonietta.casadei@uniroma1.it (M.A.C.)

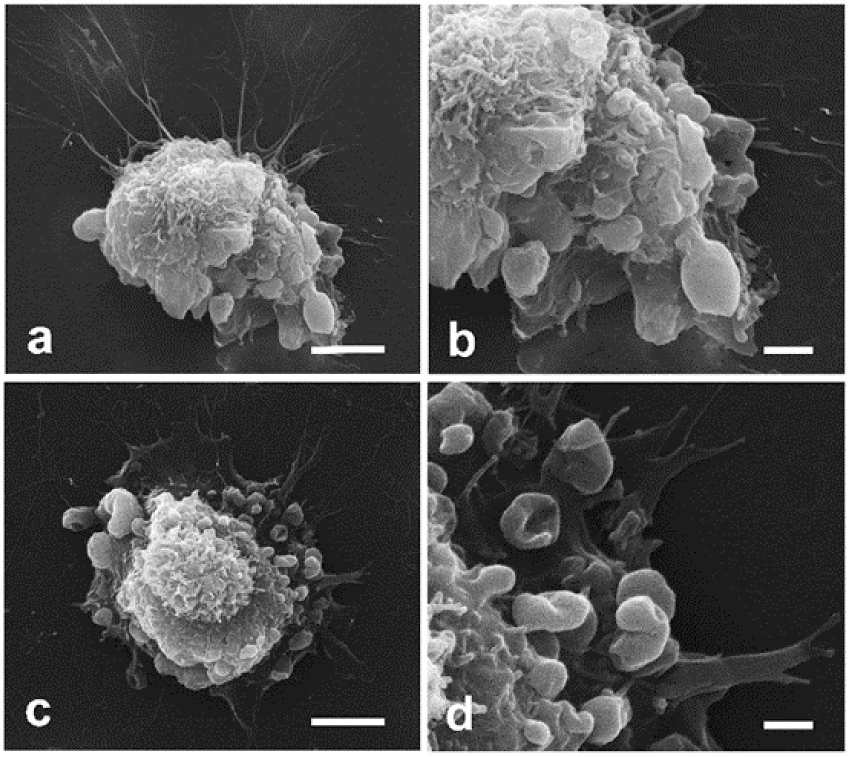

Figure S1. Scanning electron microscopy of apoptotic Caco-2 cells. The apoptotic features as reduction of the cell volume, a roundish shape, detachment from the surface and apoptotic membrane blebbing are clearly evident after treatment with LEO and NanoLEO. (**a**) LEO treated cell; (**b**) Particular of apoptotic membrane blebbing of LEO treated cell; (**c**) NanoLEO treated cell; (**d**) Particular of apoptotic membrane blebbing of NanoLEO treated cell. Bars: = (**a**), (**c**) 5 μm; (**b**), (**d**) 1 μm.
